# The tilt illusion arises from an efficient reallocation of neural coding resources at the contextual boundary

**DOI:** 10.1073/pnas.2421565122

**Published:** 2025-04-23

**Authors:** Ling-Qi Zhang, Jiang Mao, Geoffrey K. Aguirre, Alan A. Stocker

**Affiliations:** ^a^Janelia Research Campus, HHMI, Ashburn, VA 20147; ^b^Department of Psychology, University of Pennsylvania, Philadelphia, PA 19104; ^c^Department of Neurology, University of Pennsylvania, Philadelphia, PA 19104

**Keywords:** Fisher information, efficient coding, orientation estimation, fMRI, contextual modulation

## Abstract

The tilt illusion—a bias in the perceived orientation of a center stimulus induced by an oriented surround—illustrates how context shapes visual perception. Although extensively studied for decades, we still lack a comprehensive account of the illusion that connects its behavioral and neural characteristics. Here, we demonstrate that the tilt illusion originates from dynamic changes in neural coding precision induced by the surround context. We simultaneously obtained psychophysical and functional MRI responses from human subjects while they viewed gratings in the absence and presence of an oriented surround and independently extracted sensory encoding precision from their behavioral and neural data. Both measures show that in the absence of an oriented surround, encoding reflects the natural scene statistics of orientation. However, with an oriented surround, encoding precision is significantly increased for stimuli similar to the surround orientation. This local change in encoding is sufficient to predict the behavioral characteristics of the tilt illusion using a Bayesian observer model. The effect of surround modulation increases along the ventral stream and is localized to the portion of the visual cortex with receptive fields at the center-surround boundary. The pattern of change in coding accuracy reflects the surround-conditioned orientation statistics in natural scenes, but cannot be explained by local stimulus configuration. Our results suggest that the tilt illusion naturally emerges from an adaptive coding strategy that efficiently reallocates neural coding resources based on the current stimulus context.

## Significance

The tilt illusion is a prominent example of how vision is influenced by sensory context, in which the perceived orientation of a central stimulus is altered by its surround. Previous research has focused on how context affects the response properties of individual neurons. Here, we present a comprehensive explanation of the tilt illusion at both behavioral and neural levels, showing that it arises from changes in neural encoding precision induced by the surround context. We provide a normative account of these encoding changes by linking neural representations to statistical properties of natural scenes. Our findings suggest that the tilt illusion is a natural consequence of the visual system optimizing its coding capacity based on spatial context.

Human perception is significantly influenced by sensory context. A classic example is the tilt illusion, in which the perceived orientation of a central stimulus is altered by the orientation of its surround (1). Previous investigations of the tilt illusion have primarily focused on how surround context alters the response properties of individual neurons. For example, orientation-selective neurons in the early visual cortex both suppress their responses close to, and shift their tuning preferences away from, the contextual orientation (2–5). More broadly, studies of the nonclassical receptive field have shown that neural responses to stimuli within the receptive field can exhibit complex dependencies on content outside it (6, 7). These surround-dependent changes in neural response have been attributed to dynamic gain control (8), which removes redundancies in neural signals through a divisive normalization mechanism (4, 9).

How these observations at the level of single neurons give rise to perceptual behavior remains an open question. Addressing this requires simultaneous recordings from large populations of sensory neurons under contextual modulation to characterize changes in neural representation at the population level. It also requires an understanding of how these changes are then interpreted by neurons at different downstream processing stages. Previous modeling work has approached this problem by simulating neural population responses and assuming simple linear decoders (10, 11). More broadly, we lack a unified framework that accounts for the tilt illusion at the observer level, while quantitatively linking neural population responses to perceptual behavior.

Here, we provide this synthesis by studying the tilt illusion with simultaneous measurements of psychophysical behavior and neural activity using functional MRI (fMRI). We analyzed both the behavioral and neural data within a unified information-theoretic framework. Specifically, we extracted the Fisher Information (FI) of orientation encoding as a measure of coding accuracy. We computed “behavioral FI” based on a lawful relationship between FI and the bias and variance of psychophysical stimulus estimates (12, 13). We also obtained “neural FI” from early visual areas by fitting voxel-wise probabilistic encoding models to the fMRI data (14, 15). Within this framework, behavioral and neural measures of encoding accuracy can be directly compared to each other, and (via the efficient coding hypothesis) to orientation priors measured from natural scenes (16, 17). Furthermore, we can leverage the retinotopic organization of the occipital cortex to determine where potential changes arise in neural encoding accuracy relative to the spatial structure of the stimulus.

Our results show that neural and behavioral measures of encoding accuracy are qualitatively consistent across all conditions. In the absence of an oriented surround, orientation encoding precision reflects the orientation statistics of natural scenes. However, in the presence of a spatially oriented surround, encoding accuracy significantly increases at the surround orientation in a way consistent with the conditional orientation statistics of spatially adjacent regions of natural scenes. These changes in encoding cannot be explained by local effects of stimulus configuration [i.e., “vignetting” (18, 19)]. We further demonstrate that the change in encoding precision measured at the neural level is sufficient to fully predict perceptual reports of the tilt illusion based on a Bayesian observer model of orientation estimation (20). Finally, we find that the change in neural encoding occurs at the boundary between the center and surround of the stimulus, with its magnitude increasing along the ventral visual hierarchy. Our results support the notion that the tilt illusion arises from an efficient reallocation of coding resources based on stimulus context.

## Results

**Fig. 1.**
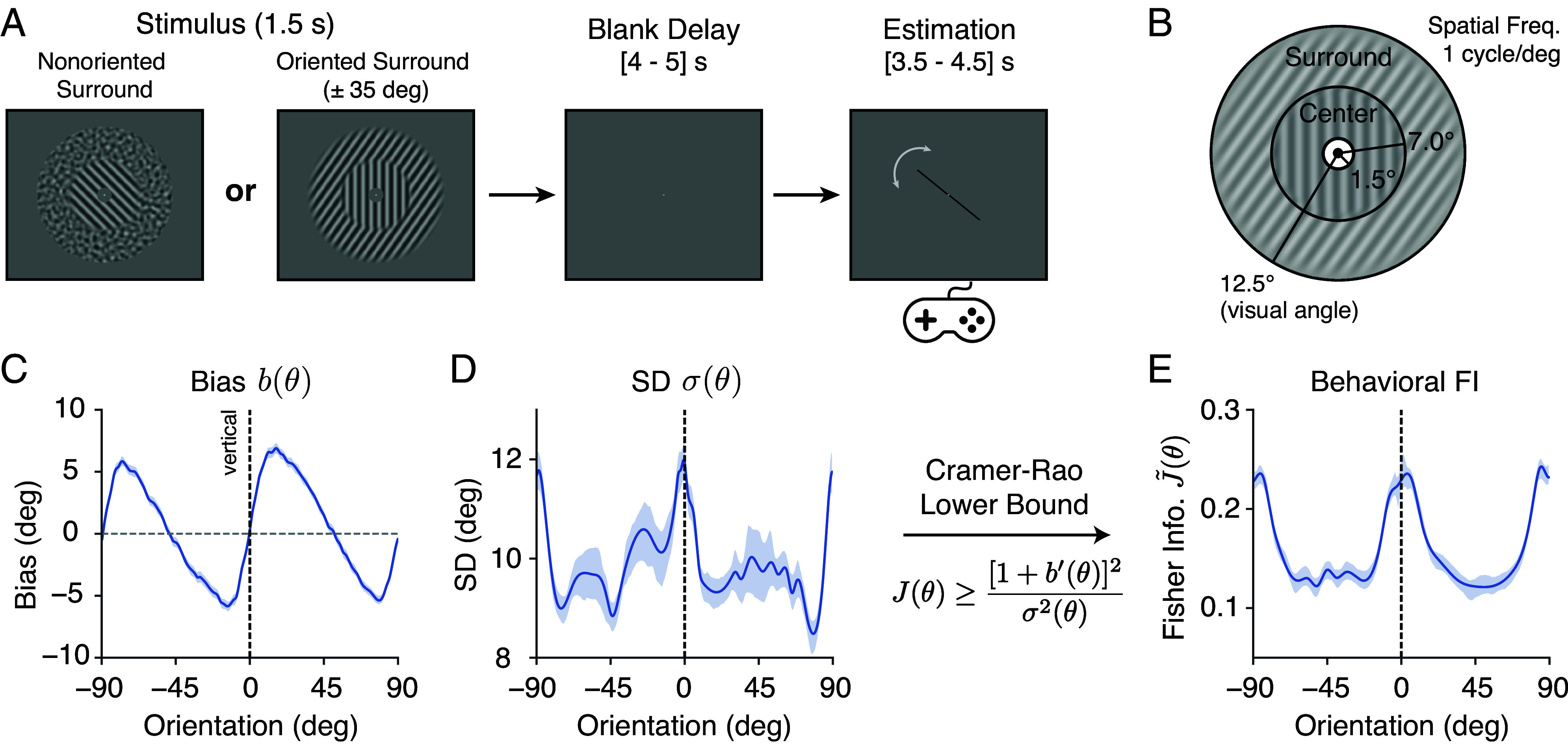
Experimental design and behavioral data analysis. (*A*) Subjects (n = 10) performed a delayed orientation estimation task across 1,200 trials during fMRI. Center gratings were presented within an annular surround of either a nonoriented, spatially filtered noise pattern, or one of two oriented gratings (±35^°^ off vertical). (*B*) Stimulus configuration. The center extends from 1.5 to 7^°^ of visual angle in radius. The surround extends from 7 to 12.5^°^ radius. (*C*–*E*) Behavioral data for the combined subject in the nonoriented surround condition; see *SI Appendix*, Fig. S9 for individual subjects. (*C*) Estimation bias b(θ) as a function of stimulus orientation. (*D*) SD σ(θ) of the estimates as a function of stimulus orientation. (*E*) Fisher Information [square root, normalized; denoted as $\tilde{J}(\theta )$] quantifying orientation encoding precision, derived from estimation bias and variance using the Cramer-Rao Lower Bound (*Methods*). Here, $b^{\prime }\left(\theta \right)$ denotes the derivative of bias w.r.t. θ. Shaded areas indicate ±SEM.

We conducted a delayed orientation estimation task inside the fMRI scanner while measuring blood-oxygen-level-dependent (BOLD) activity (Fig. 1*A*). Trials began with a 1.5 s presentation of a grating stimulus. The grating was presented within an annular surround consisting of either nonoriented noise (baseline), or a grating with one of two fixed orientations (±35^°^ off vertical). Fig. 1*B* shows the spatial configuration of the stimulus. Following a brief, blank delay, a probe stimulus (line) appeared, and subjects were asked to rotate the probe using a two-button response pad to match their perceived orientation of the grating. Every block of the fMRI acquisition consisted of 20 trials in one of the three fixed surround conditions. Surround conditions were randomized and counterbalanced across acquisitions. Each subject completed a total of 1,200 trials across six sessions of data collection.

### Measuring Orientation Encoding Accuracy from Behavior.

We first examined the perceptual behavior of subjects in the nonoriented surround (baseline) condition. Fig. 1*C* depicts the estimation bias b(θ) as a function of the center orientation. Estimates exhibited a well-known oblique bias, i.e., the perceived orientation of the grating was biased away from cardinal (i.e., vertical and horizontal) orientations (13, 21–23). For example, when the center was slightly rotated clockwise (positive) from the vertical, the bias was positive, indicating that subjects perceived the orientation to be even more clockwise. The SD of the estimates σ(θ) was also not uniform across orientations but higher at cardinal compared to oblique orientations (Fig. 1*D*).

We took advantage of the Cramer-Rao Lower Bound (CRLB) to quantify encoding accuracy based on these behavioral measures (13, 24). The CRLB describes the bounded, lawful relationship between estimation bias b(θ) and variance $\sigma^{2}\left(\theta \right)$ of an estimator, and the FI J(θ) of its sensory encoding as follows:

[1] $$J\left(\theta \right)\geq \frac{{\left[1+b^{\prime }\left(\theta \right)\right]}^{2}}{\sigma^{2}\left(\theta \right)},$$

where $b^{\prime }\left(\theta \right)$ is the derivative of bias b w.r.t. orientation θ.

We have previously demonstrated (13) that equating this lower bound with FI requires only the weak and common assumption that the estimator and the subsequent response process (i.e., motor control) are not corrupted by stimulus-dependent noise.

Eq. **1** allowed us to extract encoding accuracy in terms of FI from subject responses without the need to assume a specific decoding model. We found that orientation encoding in the nonoriented surround condition was nonhomogeneous (Fig. 1*E*): FI was highest at the cardinal orientations, and lowest at the obliques. Because FI is inversely related to discriminability (11, 12, 25), our result is consistent with previous measurements of orientation discrimination thresholds, which have consistently shown lower thresholds at cardinal than oblique orientations (20, 22, 26).

### Measuring Orientation Encoding Accuracy from Neural Responses.

**Fig. 2.**
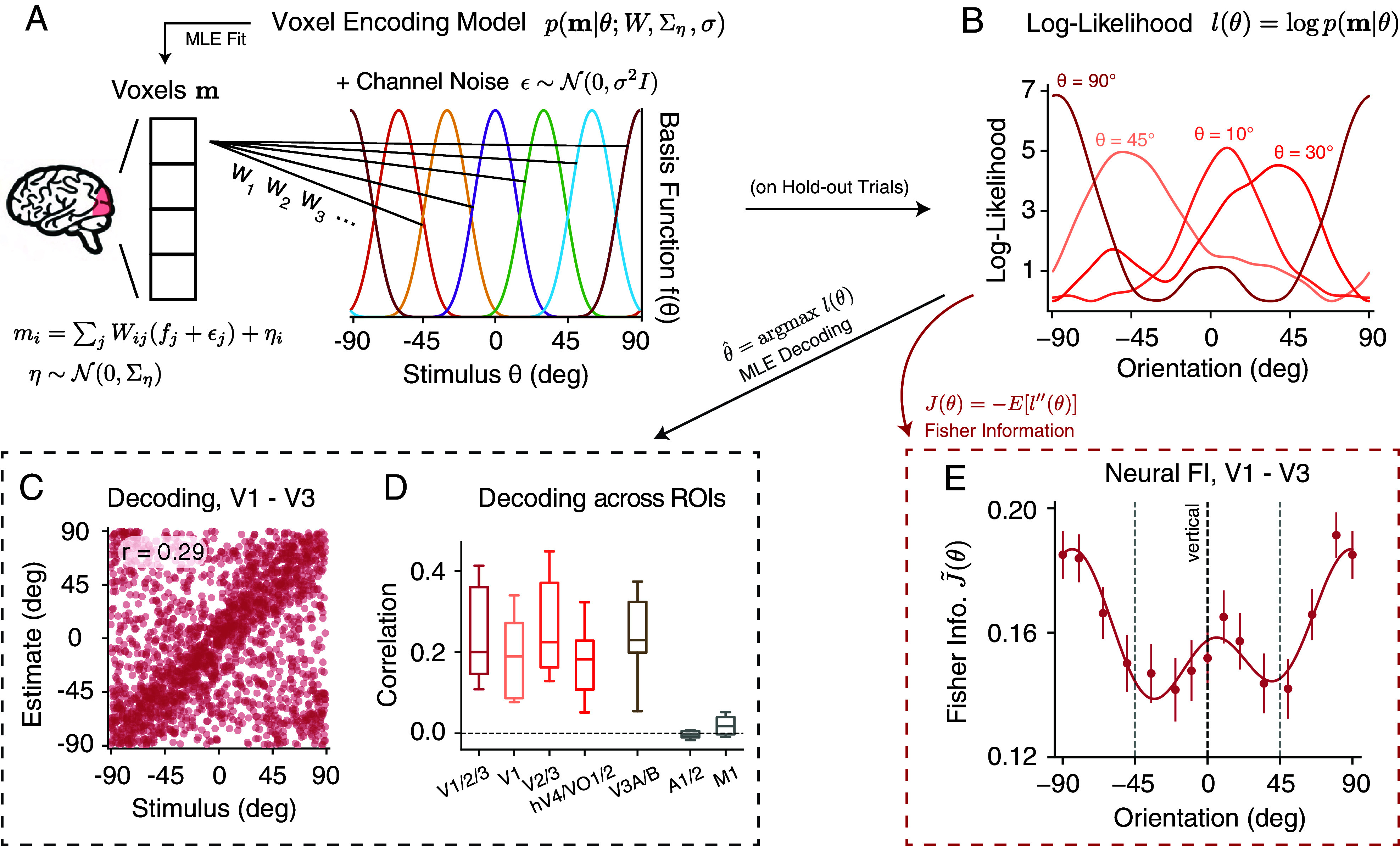
Neural data analysis. (*A*) We quantified the voxel responses m using a population encoding model (14, 15), denoted as p(m|θ). The normalized activity for each voxel $m_{i}$ was modeled as a weighted sum of responses from a set of basis functions. We assumed that each basis function was corrupted by channel noise ϵ. Additional variability of each voxel was modeled by residual noise η. Model parameters were obtained by fitting the voxel data using a two-stage procedure. (*B*) The orientation log-likelihood of the model l(θ)=logp(m|θ) was obtained based on hold-out trials. (*C*) The orientation of the stimulus presented on each trial can be decoded as the orientation with highest likelihood, thus $\hat{\theta }=\;\text{argmax}\;l\left(\theta \right)$. Here, we show a scatter plot of the stimuli orientation (x-axis) vs. the decoded orientation (y-axis) from the early visual cortex (V1 to V3), for all trials in the nonoriented surround condition across five subjects. The decoding performance r is quantified as the circular correlation between stimulus and the MLE estimates. (*D*) Decoding (circular) correlation from different ROIs in the visual cortex and two control ROIs (auditory cortex and primary motor cortex). The box extends from the first to the third quartile of the average decoding correlation of all trials across individual subjects, with the center line at the median. The whiskers indicate the farthest data point. (*E*) Fisher information (FI) of neural encoding was defined as the negative average second derivative of the log-likelihood, $J\left(\theta \right)=-E[l^{\prime \prime }\left(\theta \right)]$. Shown is the neural FI (normalized, square root) of the early visual cortex (V1 to V3) for the combined subject in the nonoriented surround condition. Error bars indicate ±SEM. See *Methods* for details.

Next, we extracted neural measures of encoding accuracy from BOLD fMRI signals recorded during the delay period. We defined regions of interest (ROIs) based on separately measured retinotopic maps for each subject. Voxels from different visual areas within the visual eccentricity range of the grating stimulus were selected. We first fit a voxel-wise probabilistic encoding model (14, 15) to the normalized BOLD activity, averaged across a time-window from 4 to 8s after stimulus onset. Separate models were fit for each ROI, each subject, and each surround condition. The encoding model describes the activity of each voxel as a weighted sum of responses from a set of basis functions. Additionally, the model incorporates two sources of Gaussian noise: tuning-dependent noise and voxel-wise residual noise. Collectively, this model defines a multivariate voxel population encoding model p(m|θ) (Fig. 2*A*; see *Methods*, Eq. **9**).

For any given pattern of voxel BOLD activity m, the encoding model defined a sensory log-likelihood function l(θ)=logp(m|θ) (Fig. 2*B*). Previous studies have used the likelihood function to decode both the stimulus and its associated uncertainty for orientation (14), motion direction (27), and working memory content (28) from BOLD activity. While not our main focus, we found that we could reliably decode orientation from a range of visual areas, but not from two control areas (auditory and motor cortex; Fig. 2 *C* and *D*). Decoding performance was comparable between the three surround conditions but tended to be slightly higher for the oriented surround (*SI Appendix*, Fig. S1).

We used the log-likelihood function to derive neural FI. For each trial, we calculated the negative second derivative of the log-likelihood function at the stimulus orientation. The neural FI is the expected value of this negative second derivative. To compute the FI for the combined subject, we aggregated the results from individual subjects and calculated the average over a 25-degree window centered at different orientations (Fig. 2*E*; see *Methods* for details). Consistent with behavioral FI (Fig. 1*D*), neural FI in the early visual cortex (V1 to V3) was highest around cardinal and lowest at oblique orientations in the nonoriented surround condition.

### Orientation Encoding Reflects Natural Scene Statistics.

In the previous sections, we demonstrated that in the nonoriented surround condition both behavioral and neural measures of FI show similar, nonuniform patterns as a function of orientation. What is the origin of this nonhomogeneous encoding pattern, and in particular, the emphasis for cardinal orientations? The efficient coding hypothesis suggests that there is a direct relationship between stimulus prior p(θ) and encoding FI for neural codes that aim for an optimal stimulus representation given resource constraints (16, 17, 29):

[2] $$p\left(\theta \right)\propto \sqrt{J(\theta }).$$

**Fig. 3.**
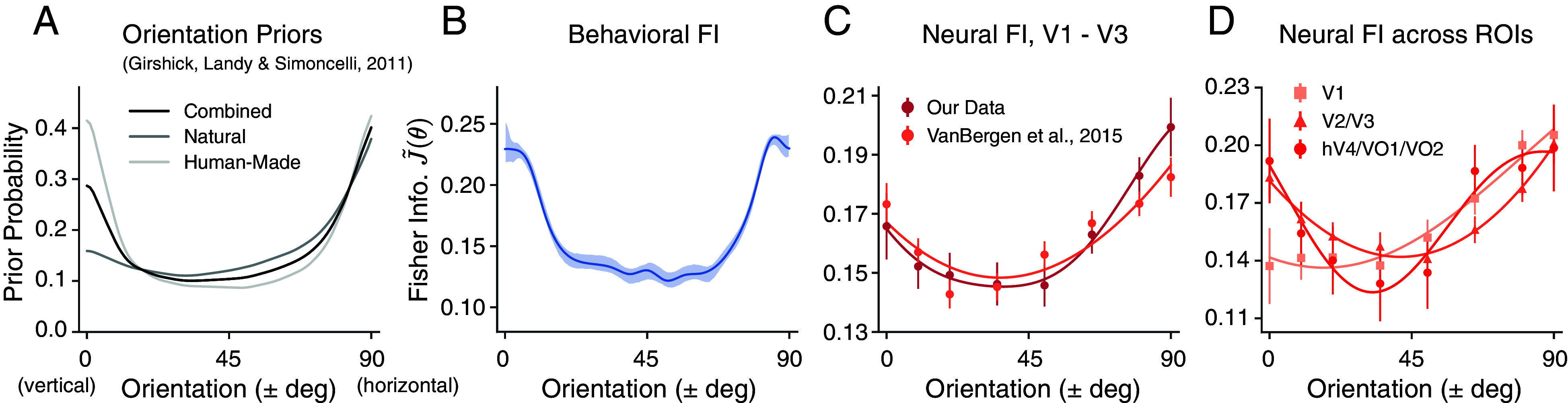
Comparison between the orientation priors derived from photographic images, the behavioral FI, and the neural FI in the nonoriented surround condition. For all panels, we assumed vertical symmetry and combined the data from corresponding counterclockwise and clockwise orientations. (*A*) Orientation priors measured in different visual environments, reproduced from ref. 31. (*B*) Behavioral FI calculated from the estimation data (same as in Fig. 1*D*). (*C*) Neural FI in the early visual cortex calculated from the voxel encoding model for our data (Fig. 2*E*), and another dataset (14). (*D*) Neural FI for different visual areas. The data plotted are for the combined subject; shaded area and error bars indicate ±SEM. All FI curves represent the normalized, square root of Fisher information, $\tilde{J}\left(\theta \right)$.

Thus, the normalized, square root of FI, $\tilde{J}\left(\theta \right)$, can be interpreted as the inferred orientation prior assuming an efficient neural encoding (30), which allows for a direct comparison with other estimates of the orientation prior. For example, Fig. 3*A* shows the statistics of local visual orientation computed over large subsets of photographic images containing more or fewer natural objects (31). In both natural and human-made environments, the prior probability of cardinal orientations is higher than that of oblique orientations.

Consistent with the efficient coding hypothesis, the behavioral FI pattern we found in the nonoriented surround condition resembles these environmental priors (Fig. 3*B*). Furthermore, we observed the same qualitative match with the neural encoding accuracy (neural FI) in the early visual cortex (V1 to V3). This was confirmed by an identical analysis of a previously reported dataset (14) (Fig. 3*C*). Last, to examine how the orientation prior is represented across different visual areas, we obtained the neural FI separately for three groups of ROIs, organized along the visual ventral hierarchy (Fig. 3*D*). We found a strong cardinal emphasis in areas V2 and V3, and hV4 and VO1/2; the neural FI in these areas was most similar to the orientation prior in natural scenes.

### Surround Selectively Increases Encoding Accuracy.

**Fig. 4.**
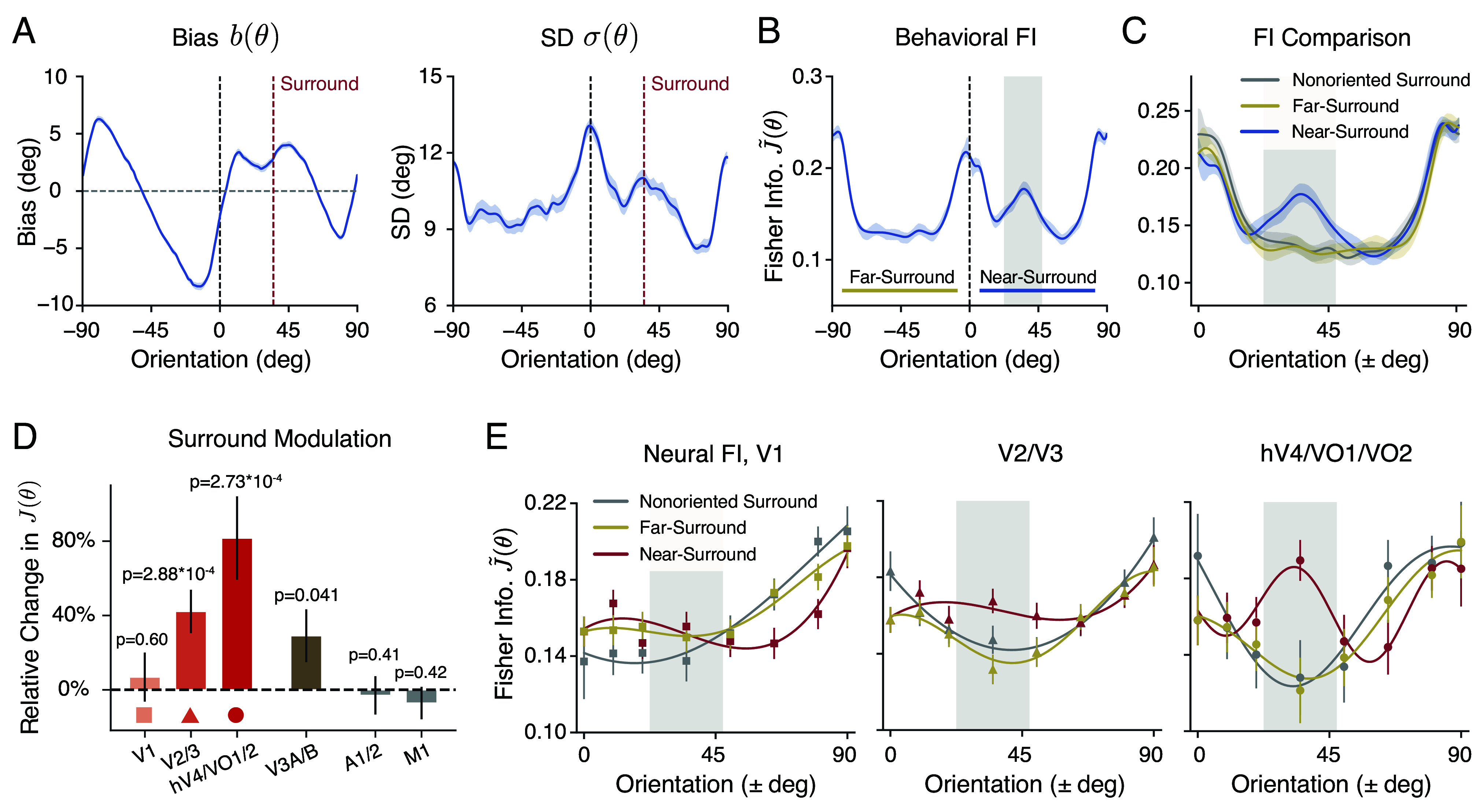
Orientation encoding in the tilt illusion. We analyzed the behavioral and neural data in the oriented surround condition in the same way as in the nonorientated condition (combined subject). (*A*) Estimation bias b(θ) and SD σ(θ) as a function of the orientation of the center grating. (*B*) Behavioral FI, calculated from estimation data. “Near-surround” refers to the 90-degree orientation range (vertical to horizontal) on the side of the surround orientation, and “far-surround” refers to the 90-degree range on the side opposite to the surround orientation. Gray-shaded area indicates a 25-degree window (between 22.5 and 47.5^°^) centered at the surround orientation. (*C*) Comparison of the behavioral FI between near-surround and far-surround orientations in the oriented surround condition and the nonoriented (baseline) condition. (*D*) The relative percentage change in neural FI within the gray-shaded area, for different ROIs in the visual cortex, and two control ROIs. (*E*) Comparison of neural FI along the visual ventral stream. Shaded areas and error bars indicate ±SEM. See *SI Appendix*, Fig. S3 for the unnormalized FI. See *SI Appendix*, Figs. S9 and S10 for behavioral and neural FI of individual subjects.

We next examined the behavioral and neural data from the oriented surround conditions (i.e., the tilt-illusion; Fig. 4). As in the previous analysis, we assumed symmetry around the vertical meridian. We thus aggregated the data measured from the two symmetric surround orientation conditions (i.e., positive angles for the +35^°^ surround and negative angles for the −35^°^ surround). We denote the 90-degree orientation range (vertical to horizontal) containing the surround orientation as “near-surround,” and the opposite range as “far-surround,” respectively.

The oriented surround altered both the bias and variance of the subjects’ orientation estimates of the center grating, especially for orientations close to the surround (Fig. 4*A*; compared to Fig. 1 *C* and *D*). These changes are consistent with well-known characteristics of the tilt-illusion (1, 32), showing a strong repulsive bias near the surround orientation and a subtle attractive bias further away (*SI Appendix*, Fig. S2*A*). We again used Eq. **1** to extract behavioral FI. We found that the oriented surround leads to a significant increase in encoding precision close to the surround orientation, while the overall FI pattern—in particular for “far surround” orientations—remains unchanged (Fig. 4*B*). This is especially apparent in the direct comparison of the behavioral FI for both the near- and far-surround orientation range alongside the FI for the nonoriented surround (baseline) condition (Fig. 4*C*).

The same characteristic change in orientation encoding is observed in the neural measurements. We derived the neural FI by fitting a separate set of voxel encoding models to the fMRI data collected in the oriented surround condition. We found a significant effect of surround modulation on encoding accuracy in several areas of the early visual cortex. Consistent with the behavioral measure, neural FI is substantially increased within a narrow window near the surround orientation compared to the baseline condition. The magnitude of this effect increases along the visual ventral stream with an apparent peak in the combined area hV4/VO1/VO2 (Fig. 4*D* and *SI Appendix*, Figs. S3 and S4). Particularly in these later areas, the encoding pattern (Fig. 4*E*) is remarkably similar to the behavioral FI (Fig. 4*C*).

**Fig. 5.**
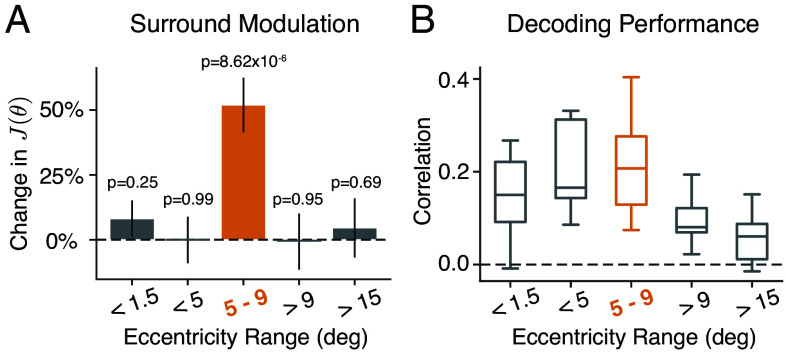
Surround modulation for ROIs at different stimulus eccentricities. Voxels from within areas V1 to V3 were selected based on the center and size of their pRFs (*Methods*). (*A*) The relative change in neural FI with respect to the baseline near the surround orientation for ROIs at different eccentricities. Error bars indicate ±SEM. (*B*) Average decoding (circular) correlation for all subjects using voxels with ROIs at different stimulus eccentricities.

We further examined how the change in neural encoding precision depended upon which part of the stimulus was encoded. We computed neural FI for different subsets of voxels with different eccentricity ROIs based on the center and size of their population receptive fields (pRF; see *Methods*). We found that encoding precision computed for voxels with pRFs exclusively within the surround region did not exhibit any effect of surround modulation (Fig. 5*A*; >9^°^ and >15^°^). Similarly, encoding precision extracted for voxels with pRFs strictly within the center remained unaffected by the surround (Fig. 5*A*; <5^°^ and <1.5^°^). In contrast, encoding precision for voxels at the contextual boundary was strongly modulated (Fig. 5*A*, 5 to 9^°^). This suggests that changes in neural FI are driven by modulation of the center encoding through interactions between center and surround regions and that these interactions are spatially localized to the area close to the center-surround contextual boundary. Notably, the eccentricity band for which the change in FI was observed did not necessarily correspond to the region of the stimulus for which the strongest orientation decoding was found (Fig. 5*B*). Finally, in a similar set of analyses but using ROIs defined based on the polar angle assignment of the voxels, we found that the surround modulation is stronger in voxels spatially aligned with the direction of the surround orientation, compared to those oriented orthogonally to it (*SI Appendix*, Fig. S5).

### Changes in Neural Coding Precision Predict the Tilt Illusion.

**Fig. 6.**
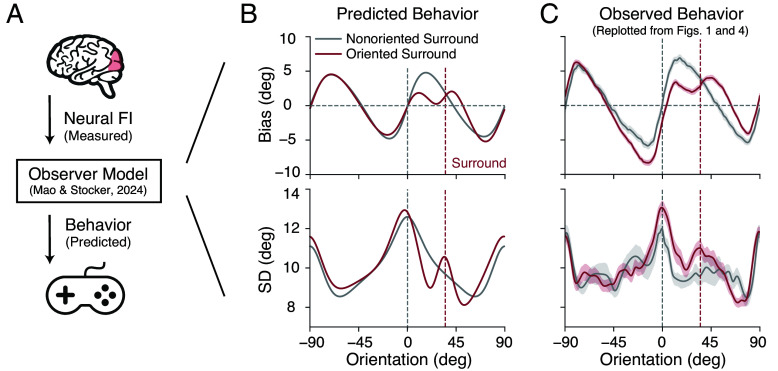
Predicting the tilt illusion from the neurally measured encoding precision. (*A*) We used the extracted neural FI of areas hV4/VO1/VO2 in the nonoriented surround condition (baseline) and oriented surround condition to predict subjects’ behavior (i.e., mean and SD of their orientation estimates) based on a state-of-the-art Bayesian observer model for orientation estimation (20). Data and predictions are for the combined subject. (*B*) Predicted bias and SD. (*C*) Measured estimation bias and SD, replotted from Figs. 1 and 4. Gray curves indicate the baseline and red curves indicate the oriented surround condition. Shaded areas represent ±SEM. See *SI Appendix*, Fig. S6 for further analysis of the model.

So far, we have established a close correspondence between behaviorally and neurally estimated changes in encoding accuracy. We have shown that the tilt illusion coincides with a consistent, characteristic increase in encoding precision for orientations similar to the surround orientation. To better understand the role of these encoding changes, we tested whether the observed neural changes in FI can directly predict the psychophysical reports of the tilt illusion (Fig. 6*A*). We employed a recently developed Bayesian observer model for orientation estimation (20). The model assumes that encoding is efficient (Eq. **2**), which jointly constrains the likelihood function and prior distribution of the model. Thus, for any given function of the encoding precision (e.g., measured as FI) the model is tightly constrained and able to make quantitative predictions of subjects’ orientation estimates.

We set the encoding precision of the model to reflect the neural FI measured for areas hV4/VO1/2 (Fig. 4). We first used the data from the baseline condition to determine the remaining free global parameters of the model (e.g., overall sensory noise). Then, we updated the modeled encoding precision and the prior to match the neural FI measured for the surround condition. The model output provided predictions of the perceptual bias and SD in the absence and presence of an oriented surround (see *Methods* for more details). As shown in Fig. 6*B*, the model successfully recapitulates the pattern of estimation bias and SD in the baseline condition (gray lines), which confirms the result of the previous study (20). Moreover, it also accurately predicts the characteristic changes in bias and SD observed in the tilt illusion (red lines). This includes the repulsive bias near the surround orientation (as indicated by the positive slope of the bias curve; one of the most prominent features of the tilt illusion), as well as the sharp, local increase in SD.

Note that a key assumption of the model is that orientation reports are the result of a holistic inference process that jointly operates at low- and high-level representations of the stimulus (i.e., stimulus orientation, but also orientation categories, such as vertical and horizontal orientations). Here, we assumed that the surround orientation provides an additional category boundary. We verified that incorporating both the adaptive change in encoding precision and the categorical boundary at the surround are necessary for the model to correctly predict the tilt illusion *SI Appendix*, Fig. S6.

### Surround Modulation Reflects Changes in Neural Response Characteristics.

**Fig. 7.**
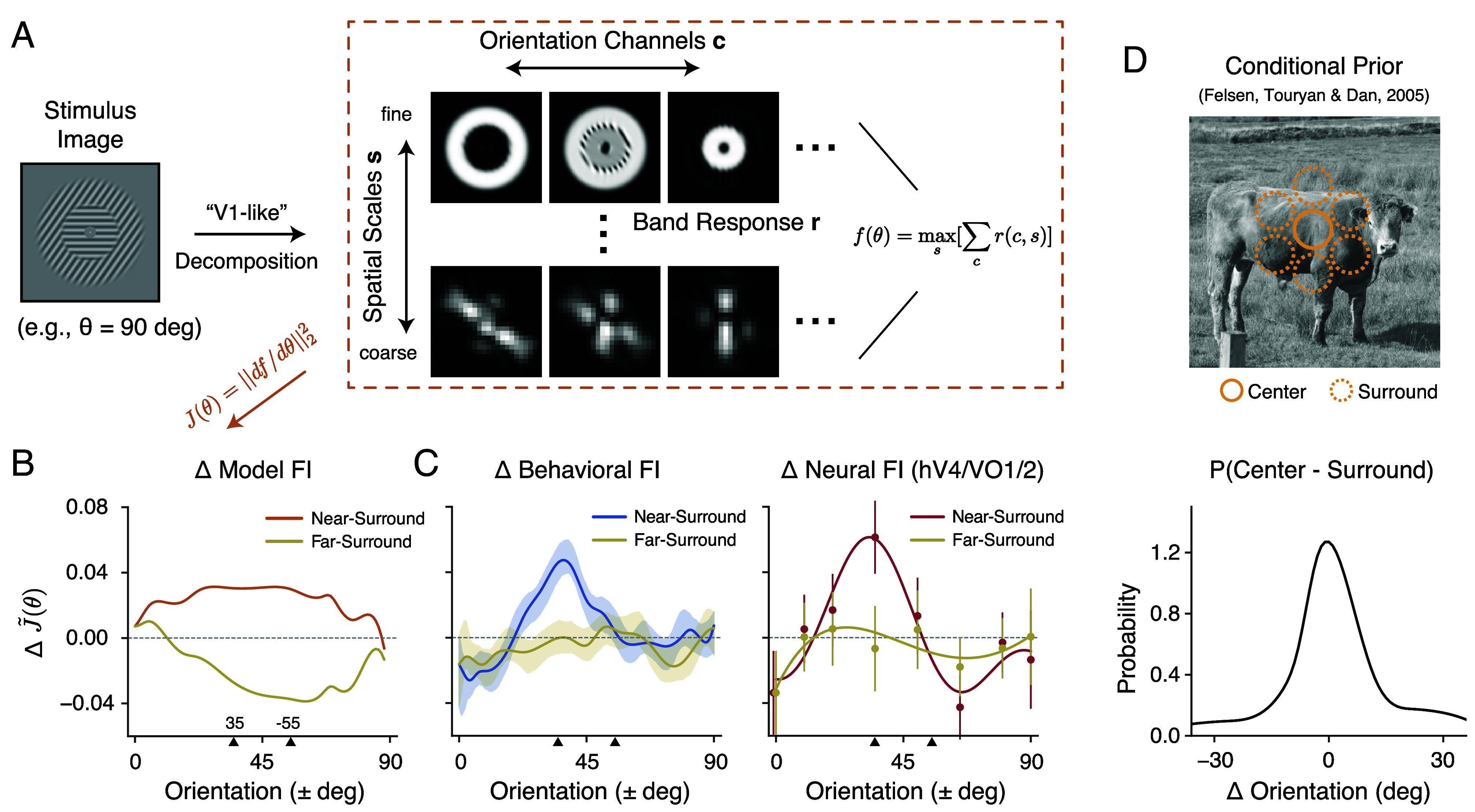
The effect of surround modulation cannot be explained by spatial stimulus configuration but is consistent with natural scene statistics. (*A*) We simulated a “retinotopic map” of voxel responses f(θ) by averaging across orientation channels, and selecting the spatial scale with the largest orientation information in a steerable pyramid decomposition r(c,s) (*Methods*). (*B*) Changes in encoding FI ($\Delta \tilde{J}\left(\theta \right))$ between stimuli with oriented surround compared to stimuli with nonoriented surround (baseline) condition based on the steerable pyramid voxel encoding model. The two ticks on the x-axis denote the surround orientation (+35^°^) and the orientation orthogonal to the surround (−55^°^). (*C*) Changes in behavioral FI and neural FI with surround modulation compared to the baseline condition. (*D*) Probability distribution of the angular difference in orientation between the center and surround regions of natural images (adapted from ref. 34). Shaded area and error bars indicate ±SEM.

We have demonstrated that the tilt illusion arises from changes in orientation encoding in the presence of an oriented surround context. What is the origin of these changes in encoding accuracy? One possibility is that the addition of an oriented surround naturally leads to increased coding accuracy near the surround orientation because of the nonlinear processing of the visual system. In this case, there are no changes in the response properties of the sensory neurons, and the difference observed in encoding accuracy is purely due to the nonisotropic spatial configuration of the stimulus. The potential effect of spatial configuration is closely related to the issue of “stimulus vignetting” (18, 19), in which the arrangement of the stimulus and its aperture can result in additional signals for orientation encoding. To quantify the changes in the measured encoding FI that arise solely due to differences in stimulus configuration (i.e., nonoriented vs. oriented surround) in the absence of changes in neural response properties, we implemented an image-computable voxel encoding model (19). The model first applies a decomposition of the stimulus image, generating multiple bands of filter responses with varying orientations and spatial frequency selectivity [a steerable pyramid (33)]. A map of simulated voxel responses can be obtained by combining these bands. Although each voxel in this construction is not orientation-selective, the pattern of responses across voxels as the grating rotates can still provide information about grating orientation. We again quantify this information using FI (Fig. 7*A*; see *Methods*).

For stimuli with a nonoriented surround, the encoding FI was nonzero (*SI Appendix*, Fig. S7), reflecting the vignetting effect reported by Roth et al. (19). Note, however, that the FI is uniform across orientation because any effect of stimulus configuration in the nonoriented condition is isotropic by design. Next, we calculated the changes in FI for stimuli in the oriented surround condition (Fig. 7*B*). We found that the oriented surround elicited a broad increase in FI for the near-surround orientations compared to the baseline condition. At the same time, it also caused a broad decrease in FI for the far-surround orientations, with the lowest point at the orientation orthogonal to the surround (−55^°^). This pattern was different from the changes in FI we observed in both our behavioral and neural data (Fig. 7*C*), where the increase in FI was limited to a small range around the surround orientations while encoding accuracy remained essentially unchanged for far-surround orientations. Thus, the spatial configuration of the stimulus alone cannot explain the measured changes in encoding accuracy. Rather, additional surround-induced mechanisms must be at work that adaptively adjust the neural representation of stimulus orientation, similar to what has been observed at the level of individual neurons (2–6).

But why should the visual system actively increase encoding precision close to the surround orientation? We again turn to the efficient coding hypothesis, which suggests that the increase in FI should correspond to a local increase in the probability of those orientations. Spatial structures in adjacent regions of natural images are indeed correlated (35). Therefore, the observation of a specific surround orientation indicates a marked increase in the probability of the center orientation being similar to that of the surround (Fig. 7*D*). We found that the change in encoding FI (Fig. 7*C*) closely resembles the probability distribution of orientation difference between center and surround regions in natural images. We thus conclude that surround modulation is consistent with an efficient reallocation of encoding resources guided by context-specific statistical regularities.

## Discussion

Our study reveals the sensory origin of the well-known tilt illusion. Based on concordant measures of encoding precision from behavioral and neural data, we demonstrate that the presence of an oriented surround causes a dynamic change in neural encoding precision, such that sensory representations remain optimized for both the long-term as well as the local surround-conditioned statistics of orientations found in natural scenes. The strength of the neural encoding change increases along the ventral visual stream and is spatially localized to the boundary between the center and contextual surround. Furthermore, we show that the reported encoding change is sufficient to predict subjects’ behavior in the tilt illusion using a state-of-the-art Bayesian observer model of orientation estimation that assumes efficient sensory encoding. Our findings support the notion that the tilt illusion originates from a sensory system that adaptively updates its encoding characteristics according to stimulus context to maximize information capacity (10, 36–39).

We use FI as a common metric to quantify sensory encoding precision, which offers several advantages. First, it allows us to extract sensory encoding characteristics from subjects’ reports in our psychophysical orientation estimation task using a lower-bound relation between FI and estimation bias and variance (13). It also allows us to directly compare our results with discrimination threshold experiments because discrimination thresholds are inversely proportional to FI (11). Previous studies have reported discrimination thresholds with (40) and without (22, 26) spatial context that are well aligned with our results. Finally, FI enables a direct comparison of encoding accuracy derived from simultaneously recorded behavioral and neural data without the need for an explicit decoding model.

While the extracted FI quantifies how the precision of sensory encoding changes in the presence of an oriented surround, it does not specify the underlying neural mechanisms responsible for these changes (41). Previous studies have documented a diverse set of possible mechanisms at the level of neuronal tuning characteristics, including changes in response gain, tuning preference, and tuning width (5, 7, 34, 42, 43). All these changes combined and accumulated across a neural population, as well as potential noise correlations (44, 45), then determine FI at the level we have measured in our study. These changes may arise from either feedforward or feedback activity across the visual cortex, or a combination of both. While our results provide quantitative constraints for identifying the underlying neural mechanisms, additional experimental work is required to test specific mechanistic hypotheses in more detail. Such studies would likely require exploring various stimulus configurations across multiple spatial scales. Thus, future research will be necessary to clearly establish the connections between mechanisms operating at the individual neuron level and the population-wide changes in encoding precision.

Our results align with previous studies suggesting that the sensory cortex forms efficient representations of perceptual variables according to their long-term (prior) statistics in natural scenes (46, 47). For example, Harrison et al. (48) used electroencephalogram measurements and a forward encoding model to show that the tuning properties of cortical neurons can encode an orientation prior. Similarly, based on single-unit recording data, Zhang and Stocker (30) illustrated that a power-law, slow speed prior for visual motion is represented in the macaque MT cortex via a logarithmic encoding mechanism. What sets our results apart from these previous findings is that they are obtained from a joint analysis of simultaneously recorded behavioral and neural data. Whole brain fMRI recordings also allowed us to pinpoint and track the neural representation of orientation priors across the representational hierarchy of the human visual cortex.

Furthermore, we show that the context-induced changes in neural encoding ensure that the sensory representation remains efficient with regard to the natural orientation statistics conditioned on the dominant surround orientation (Fig. 5). This offers a normative understanding of context-induced changes in neural encoding, and situates computational mechanisms such as lateral inhibition and divisive gain control within a broader efficient coding framework (9). Divisive normalization is considered a fast mechanism that operates within local populations of sensory neurons (4, 7). This is consistent with previous perceptual results showing that the tilt illusion follows changes in the surround orientation up to 10 Hz (49). It is also consistent with our finding that surround modulation is spatially confined to ROIs covering the center-surround stimulus boundary. Previous behavioral studies of the tilt illusion further corroborate this by showing that stronger segmentation cues at the center-surround boundary decrease the strength of the illusion (50, 51).

Although our study was focused on characterizing the changes in sensory encoding, we demonstrate that these changes are sufficient to accurately predict subjects’ reports of their perceived tilt illusion using a recently proposed Bayesian observer model (20). The specific model currently provides the most accurate quantitative descriptions of human behavior in orientation estimation tasks. Its predictions support the crucial role of the encoding changes in creating the tilt illusion. It confirms a previous theory (4) suggesting that the tilt illusion is not the result of suboptimal inference processes but rather reflects resource-rational behavior in a statistically structured environment. It is also worth noting that the Bayesian observer model assumes that subjects’ reported orientation estimates are affected by an ordinal/categorical assessment of the stimulus, i.e., whether the orientation of the center stimulus is perceived to be clockwise or counterclockwise of the surround orientation. This suggests that the surround stimulus acts as a reference in guiding subjects’ reports, which links the tilt illusion to the broader contextual effect referred to as reference repulsion (e.g., refs. 52 and 53). It also implies that the bias in the reported orientation estimates may be in part nonperceptual, arising from downstream decision processes. As a result, the repulsive biases in subjects’ reported estimates may exaggerate the actual perceptual distortions they experience during the tilt illusion.

Our current analysis focuses on group-level effects by combining data across subjects. However, previous studies have shown that the effect of the tilt illusion varies considerably across individuals (54, 55). When examining our data at the level of individual subjects, we also observe variability in both the magnitude of perceptual bias and the pattern of FI inferred from behavioral and neural data (*SI Appendix*, Figs. S9 and S10). Future research may explore whether individual differences in encoding changes can be systematically linked to variations in perceptual behavior. However, a reliable exploration of such individual differences will likely require substantially more data.

Our results offer predictions regarding other aspects of the tilt illusion. For example, previous research has shown that different surround features—such as complex textures with a broader range of orientations (56)—can also induce the illusion. We hypothesize that the perceptual characteristics of the illusion can be predicted based on the specific shape of the surround-conditioned orientation distribution in natural scenes. For instance, a surround containing a wider range of orientations is expected to predict a broader and more gradual increase in the probability of the center orientation, resulting in weaker change in encoding and, consequently, a smaller bias.

It is also worth considering the temporal homolog of the tilt illusion, namely the tilt aftereffect. In the tilt aftereffect, context is established temporally through a sequence of preceding stimuli with fixed orientation (57). The changes in orientation perception and neural tuning observed for the tilt aftereffect are remarkably similar to those found in the tilt illusion (10, 39, 58, 59). Furthermore, the conditional orientation distribution for temporally adjacent stimulus is very similar to that of spatial contexts, also peaking at the dominant orientation of the context (10, 39). Therefore, we predict similar changes in encoding precision for the tilt aftereffect as we have reported here for the tilt illusion. We have indeed recently shown that this is the case based on psychophysical threshold measurements (39). It will be intriguing to validate this further using fMRI data and investigate the extent to which the increase in contextual modulation along the visual ventral stream is also observed for the tilt aftereffect.

Finally, we presented a methodological framework that can be used for testing and understanding contextual effects involving other stimulus features, including shape [e.g. the Ebbinghaus illusion (60)], motion (61), color (62, 63), and face perception (64). We speculate that these phenomena all originate from adaptive changes in sensory representation that reflect the context-conditioned statistics in natural visual environments.

## Methods

### Experiment.

This study was approved by the University of Pennsylvania Institutional Review Board in accordance with the Declaration of Helsinki, and all subjects provided a written consent.

#### Procedure.

Subjects (n = 10) performed a delayed orientation estimation task conducted in the fMRI scanner. All subjects had normal or corrected to normal visual acuity. On each trial, a 2 s initial delay was followed by the presentation of an oriented grating stimulus for 1.5 s. The grating stimuli were presented within an annular surround of either nonoriented noise, or gratings with one of two fixed orientations (±35^°^ off vertical). After a blank delay period of 4 to 5 s, a line probe appeared, and subjects used a two-button response pad to rotate the probe to report their orientation estimates. The line probe remained on the screen for a duration between 3.5 and 4.5 s (uniformly sampled). The blank delay period was configured such that the total time of delay and response was 8.5 s. Stimulus and response tasks were created using PsychoPy (65).

Each fMRI acquisition consisted of 20 trials, with all trials within the acquisition using either the nonoriented surround, or one of the two oriented surrounds. The assignment of surround condition to acquisition order was counterbalanced within and randomized across subjects. Over six sessions of fMRI scanning, subjects completed a total of 60 acquisitions, resulting in 1,200 trials (400 trials for each surround condition).

#### Stimulus.

Subjects viewed stimuli on a computer monitor positioned at the end of the scanner bore via an angled mirror mounted on the head coil. Each stimulus consisted of a mid-gray central region with a radius of 1.5^°^, and a fixation dot of 0.35^°^. An oriented center grating occupied the area between 1.5^°^ and 7^°^ radius and had a spatial frequency of 1 cycle per degree. The orientation was sampled uniformly between 0^°^ and 180^°^. Around the center grating was an annular surround extending from 7^°^ to 12.5^°^ radius. It contained either nonoriented noise, or one of the two fixed orientations (±35^°^), all with a spatial frequency matched to the center (1 cycle per degree). The entire stimulus was contrast-modulated at 1 Hz temporal frequency with a peak contrast of 20%. See Fig. 5*A* for a schematic of the spatial configuration of the stimulus.

### Neuroimaging.

#### MRI acquisition.

Anatomical (T1w and T2w) and BOLD functional images were acquired on a Siemens 3T Prisma scanner with a 64-channel head coil at the University of Pennsylvania. For T1w images, the tfl3d1 sequence was used with 0.8 mm isotropic voxels, TR = 2,400 ms, TE = 2.2 ms, and flip angle = 8^°^. For T2w images, the SPC sequence was used with 0.8 mm isotropic voxels, TR = 3,200 ms, TE = 563 ms, and flip angle = 120^°^. The functional images were acquired with the spin echo imaging sequence epfid2d1, with 2 mm isotropic voxel size, TR = 800 ms, TE = 37 ms, and flip angle = 52^°^.

#### Retinotopic mapping.

Each subject performed an additional scanning session devoted to retinotopic mapping. The stimulus consisted of a black and white checkerboard pattern that contrast-reversed at 5 Hz temporal frequency. This pattern was displayed against a mid-gray background within a circular aperture 21^°^ in diameter. The bar moved along both cardinal and oblique orientations, with the sequence of bar positions played in reverse for the second half of the acquisition. Subjects were instructed to focus on a central black fixation dot throughout the measurements and to respond with a button press when the dot occasionally and briefly turned red. Each acquisition was 330 s, and each subject completed 6 acquisitions. T1w and T2w anatomical images were also acquired at the end of the retinotopic mapping session.

The retinotopic mapping data were analyzed using previously developed procedures (66). Briefly, a noise removal method based on independent component analysis was first applied to the functional measurements (67, 68). Population receptive field (pRF) maps were then produced by fitting a model that jointly estimates the voxel pRF and hemodynamic response function (69, 70). Last, the pRF estimates were combined with the cortical surface topology derived from structural measurements within a Bayesian framework to produce a final retinotopic map for each subject (71). The boundary of visual areas and the visual eccentricities of voxels were defined based on this map.

#### MRI data preprocessing.

We processed both the structural and functional data using the Human Connectome Project (HCP) minimal processing pipeline (72). This stage corrected for gradient nonlinearity, motion, and phase encoding direction in volumetric images. Subsequently, voxels were mapped onto a cortical surface template (fsaverage), with an additional 2 mm FWHM Gaussian surface smoothing applied. The resulting time series was high-pass filtered with a cutoff of 150 s to remove slow drifts in the BOLD response, and linear regression against the motion regressors generated by the HCP pipeline was used to further remove motion artifacts. To obtain the voxel activity pattern in response to each stimulus presentation, the time series for each trial within a session was first aligned based on stimulus onset, and then normalized (z-score) across the corresponding time point. Due to the temporal low-pass nature of the BOLD fMRI signal, neuronal activity is reflected with a delay, which is described by the hemodynamic response function (HRF). To account for the HRF, we defined the voxel response as the average value over a time window from 4 to 8 s after stimulus onset. We also tested an alternative procedure in which a generalized linear model was fitted to each subject to account for individual variability in the HRF, but this did not result in a significant improvement in decoding performance. In addition, our decoding analysis confirmed that the orientation signal in our decoding window was localized to the visual cortex but not other parts of the brain (e.g., motor cortex, Fig. 2*D*).

#### Region of interest.

We defined ROIs based on the retinotopic maps obtained using the procedure described above. In our primary analysis, we selected voxels with pRF centers between 1^°^ and 7^°^ of visual eccentricity, and from the following (groups of) visual areas: V1 + V2 + V3 (early visual cortex); V1 alone; V2 + V3; hV4 + VO1 + VO2; and V3A + V3B. Additionally, we established two control areas, the auditory cortex (A1 + A2) and primary motor cortex (M1) based on the cortical parcellation template produced by Glasser et al. (73). In an alternative analysis, we expanded the voxel pRF center to the range of 1^°^ to 15^°^ of visual eccentricity, covering the entire stimulus.

To understand the spatial profile of the surround modulation effect, we conducted an additional analysis in which voxels within area V1 to V3 were chosen based on their pRF center c and size σ in units of visual degrees (Fig. 5). To select voxels exclusively from within the center region, we defined two ROIs using the criteria $c+2\sigma <1.5^{{}^{\circ}}$, and $1.5^{{}^{\circ}}<c+2\sigma <5^{{}^{\circ}}$. To select voxels exclusively from within the surround region, we defined two other ROIs with $9^{{}^{\circ}}<c-2\sigma <15^{{}^{\circ}}$, and $15^{{}^{\circ}}<c-2\sigma <30^{{}^{\circ}}$. Last, voxels at the center-surround boundary were selected as $5^{{}^{\circ}}<c<9^{{}^{\circ}}$.

### Theoretical Framework.

We modeled orientation perception as an encoding–decoding process (74): Stimulus orientation θ is encoded as a noisy neural measurement m, described by the encoding model p(m|θ). Perceptual estimates $\hat{\theta }$ are then formed through a decoding process $\hat{\theta }\left(m\right)$ based on the neural measurement m. The FI of the encoding is defined as

[3] $$J\left(\theta \right)=-E[\frac{\partial^{2}}{\partial \theta^{2}}\;\text{log}p\left(m|\theta \right)\;|\;\theta ],$$

and quantifies the encoding accuracy as a function of θ. For a neural population that encodes information efficiently given limited encoding resources, there is a direct relationship between the stimulus prior distribution p(θ) and encoding accuracy J(θ) (16, 17, 75):

[2] $$p\left(\theta \right)\propto \sqrt{J(\theta }).$$

The goal of our analysis was to infer J(θ) independently from behavioral data (referred to as behavioral FI) and neural data (referred to as neural FI). We elaborate on the methods we used to derive these quantities in the sections below.

### Behavioral Data Analysis.

On each trial of the experiment, subjects produced an estimate $\hat{\theta }$ of the true stimulus orientation θ. For a given θ across trials, those estimates formed a distribution $p(\hat{\theta }|\theta )$. We denote the bias and variance of subjects’ estimates as b(θ) and $\sigma^{2}\left(\theta \right)$, respectively, both defined as a function of θ.

To obtain these quantities from response data, we first computed the response bias $\hat{\theta }-\theta$ for each trial. We then calculated the mean and variance of the bias within an 18-degree window, which was moved across the orientation range in steps of 0.5 degrees.

#### Cramer-Rao lower bound.

Given an encoding model p(m|θ) with FI J(θ), the Cramer-Rao Lower Bound (CRLB) states that for a biased estimator $\hat{\theta }\left(m\right)$ FI is bound from below (24) as

[1] $$J\left(\theta \right)\geq \frac{{\left[1+b^{\prime }\left(\theta \right)\right]}^{2}}{\sigma^{2}\left(\theta \right)}\;.$$

Here, $b^{\prime }\left(\theta \right)$ denotes the derivative of the bias b(θ) w.r.t. orientation θ. Thus, the Cramer-Rao bound specifies a lawful relationship between encoding accuracy and the bias and variance of an estimator (12, 13). To interpret Eq. **1**, we can denote g(θ) as the mean estimate $E_{p(\hat{\theta }|\theta )}\left[\hat{\theta }\right]$. We have b(θ)=g(θ)−θ, and the inequality Eq. **1** can be written as

[4] $$J\left(\theta \right)\geq \frac{{\left[1+{\left(g\left(\theta \right)-\theta \right)}^{\prime }\right]}^{2}}{\sigma^{2}\left(\theta \right)}={\left(\frac{g^{\prime }\left(\theta \right)}{\sigma (\theta )}\right)}^{2}\;.$$

For an unbiased estimator $g^{\prime }\left(\theta \right)=\theta^{\prime }=1$. In this scenario, there is an inverse relationship between J(θ) and $\sigma^{2}\left(\theta \right)$. When $|g^{\prime }\left(\theta \right)|<1$, the estimator performs a local compression, leading to a reduction in variance. Conversely, if $|g^{\prime }\left(\theta \right)|>1$, the estimator expands the local space, causing an increase in variance relative to 1/J(θ).

In our analysis, we assume the lower bound to be tight (or equally loose) for every θ. This allows us to infer FI from the measured estimation bias and variance. We have previously shown that a wide range of decoders, including those commonly used such as maximum likelihood and Bayesian decoders, all attain the lower bound (13). We independently applied CRLB to the estimation data from the nonoriented and oriented surround conditions, obtaining behavioral FI curves for the baseline (Fig. 1*D*) and the surround modulation condition (Fig. 4*B*), respectively. The SEM was estimated through a bootstrapping procedure that resampled the raw data 500 times.

Last, unless stated otherwise, we report the normalized, square root of FI throughout this article denoted as

[5] $$\tilde{J}\left(\theta \right)=\frac{\sqrt{J(\theta )}}{\int_{\theta }\sqrt{J(\theta )}d\theta }\;.$$

This facilitates the comparison of FI measured for different conditions, but also highlights the relationship between encoding precision and prior distribution as proposed by efficient coding (Eq. **2**): $\tilde{J}(\theta )$ can be interpreted as the orientation prior for which the neural coding is most efficient. The denominator, $\int_{\theta }\sqrt{J(\theta )}d\theta$, measures the amount of total encoding resources.

### Neural Data Analysis.

#### Voxel encoding model.

We modeled the voxel activity pattern m based on a previously developed probabilistic encoding model (14). We denote this model as p(m|θ). The model starts by assuming a set of basis tuning functions in orientation space:

[6] $$f_{j}\left(\theta \right)=\;\max{\left[0,\cos\left(\pi \times \frac{\theta -\phi_{j}}{90}\right)\right]}^{5},$$

where θ is the stimulus orientation in degrees, $\phi_{j}$ denotes the orientation preference of the j-th function. We use J=8 in our analysis, with the preferred orientation spaced equally between 0 and 180^°^.

The activity of each voxel $m_{i}$ was modeled as a weighted sum of the responses of the basis function:

[7] $$m_{i}=\sum_{j=1}^{J}W_{\mathrm{ij}}\left(f_{j}+\epsilon_{j}\right)+\eta_{i},$$

where W is the weight matrix. The model incorporates two sources of noise: Each basis function is affected by independent channel noise $\epsilon_{j}$ with variance $\sigma^{2}$: $\epsilon_{j}∼N\left(0,\sigma^{2}\right)$; and the residual noise in each voxel is modeled as $\eta ∼N(0,\;\Sigma_{\eta })$. The residual covariance matrix is constructed as

[8] $$\Sigma_{\eta }\left(\tau ,\rho \right)=\rho \tau \tau^{T}+\left(1-\rho \right)\text{I}\;\circ \tau \tau^{T}.$$

The diagonal terms of $\Sigma_{\eta }$ are $\tau_{i}^{2}$, which represents the residual variance of each voxel i, whereas ρ is a global correlation parameter such that the off-diagonal terms are $\rho \tau_{i}\tau_{j}$.

Together, this model defines p(m|θ) as a multivariate normal distribution:

[9] $$\begin{array}{c} p(m|\theta ;\;W,\sigma ,\tau ,\rho )=N(m;\;\mu (\theta ),\Omega )\\ \mu \left(\theta \right)=Wf\left(\theta \right),\;\Omega =\sigma^{2}WW^{T}+\Sigma_{\eta }\left(\tau ,\rho \right). \end{array}$$

#### Model fitting.

We fit separate encoding models to the voxel activity pattern obtained for every subject for each surround condition, and at each ROI. Each surround condition had 400 trials, with the number of voxels ranging from approximately 300 to under 2,000 depending on the ROI. A cross-validation procedure was employed in all cases, where the 400 trials were divided into 20 folds. One fold served as the hold-out data, while the model fitting was performed on the remaining folds. Orientation decoding and FI estimation were only conducted on the held-out data. This process was iterated until each fold had become the hold-out data once. Last, to avoid potential biases introduced by the specific choice of basis function, four different encoding models with phase-shifted tuning curves were fit, and results were obtained by averaging across them.

The parameters of the encoding model were obtained using a two-step procedure (14). The weight matrix was first estimated through ordinary linear regression. Denote matrix $X\in R^{N\times J}$ as the responses of J basis functions across N trials, and matrix $M\in R^{N\times K}$ as the activities of K voxels across N trials, we have

[10] $$\hat{W}={\left(X^{T}X\right)}^{-1}X^{T}M.$$

In the second step, the remaining noise parameters σ,τ,ρ were estimated (maximum likelihood estimate) given a fixed $\hat{W}$:

[11] $$\begin{array}{l} \hat{\sigma },\hat{\tau },\hat{\rho }=\underset{\sigma ,\;\;\tau ,\;\;\rho }{\arg\max}\;\sum_{i=1}^{N}\log p(M_{i}\text{|}\theta_{i};\;\hat{W},\sigma ,\;\tau ,\;\rho ). \end{array}$$

The encoding model was implemented in PyTorch (76), and the maximum likelihood was performed using the sequential least squares programming algorithm in Scipy (77). The model fittings are computationally expensive but can be sped up significantly on GPUs with PyTorch.

#### Neural FI.

For each trial in the held-out data with true stimulus orientation $\theta^{*}$ and voxel response $m^{*}$, the orientation log-likelihood can be defined using the encoding model fitted to training data (Fig. 2*B*):

[12] $$l\left(\theta \right)=\;\text{log}p\left(m^{*}|\theta ;\;\hat{W},\hat{\sigma },\hat{\tau },\hat{\rho }\right)\;.$$

Orientation decoding was performed using the maximum likelihood decoder $\hat{\theta }={\text{arg max}}_{\theta }\;l(\theta )$ (Fig. 2 *C* and *D*). To obtain the neural FI, we computed the negative second derivative of the log-likelihood function evaluated at $\theta^{*}$:

[13] $$j\left(\theta^{*}\right)=-\frac{\partial^{2}}{\partial \theta^{2}}l\left(\theta \right){|}_{\theta =\theta^{*}}\;.$$

This quantity $j(\theta^{*})$ is called observed FI (78) (i.e., FI for a specific sample of $m^{*}$), whereas the true FI, J(θ), is the expected value over j(θ): $J\left(\theta \right)=E_{m}\left[j\left(\theta \right)\right]$. For each condition in our experiment, we obtained 400 estimates of observed FI j(θ) across orientations. The values for J(θ) and its SEM were calculated by averaging j(θ) within a 25-degree window centered at various orientations (e.g., Fig. 2*E*).

Consistent with the behavioral data analysis, we report the normalized neural FI $\tilde{J}(\theta )$ as defined in Eq. **5**. The only exception was the calculation of the surround modulation index in Figs. 4*D* and 5*C* and *SI Appendix*, Fig. S4*A*. In these cases, we computed the difference between surround and baseline in the average, unnormalized j(θ) within a 25-degree window centered at the surround orientation (i.e., between 22.5^°^ and 47.5^°^). This difference was then converted to a percentage change relative to the average j(θ) across all orientations in the baseline. Statistical significance was assessed using an unpaired *t* test on the j(θ) samples within this 25-degree window.

### Observer Model for Orientation Estimation.

We predicted bias and SD of subjects’ orientation estimates using a recently proposed Bayesian observer model (20). In the following, we provide a compressed description of the model, and refer the reader to the original article for additional details.

The model assumes that orientation encoding is efficient based on the statistical (prior) distribution p(θ) over orientation θ in the observer’s environment (Eq. **2**). Moreover, it assumes that perception and the downstream decision and control process operate *holistically* on all levels of the representational hierarchy; here this includes a higher, categorical representation of orientation C (e.g., cardinal vs. oblique orientations) in addition to the feature level representation θ. Thus, the model assumes that based on a sensory signal m the observer infers posterior beliefs at both levels of the hierarchy, i.e., p(θ|m) and p(C|m), which then provide the information for the downstream estimation processes.

The orientation estimation task of our experiment requires the observer to adjust a probe stimulus such that its orientation matches the perceived orientation of the center grating (test) (Fig. 1*A*). The model assumes that the observer infers the posterior beliefs of both the orientation and the category for each of the two stimuli, probe and test. As the observer adjusts the orientation of the probe, they seek to report the probe orientation $\theta_{p}$ that minimizes the expectation of a joint objective $L_{\text{tot}}$ that reflects the mismatch between the two stimuli at both the feature and the category level; hence

[14] $$L_{\text{tot}}=\left(1-w\right)L_{\theta }\left(\theta ,\theta_{p}\right)+wL_{C}\left(C,C_{p}\right)\;,$$

where $L_{\theta }$ is defined as the cosine difference between the test and the probe orientation, and $L_{C}$ is a fixed cost if test and the probe stimuli fall into different orientation categories but zero otherwise.

For the model simulations (Fig. 6), encoding precision and the orientation prior used for Bayesian inference were determined by the neural FI of areas hV4/VO1/VO2 (Fig. 4) measured in the baseline (nonoriented surround) and the oriented surround condition, respectively. In the baseline condition, we closely followed the model specifications of the original study, assuming two orientation categories (clockwise and counterclockwise relative to vertical), and parameter values for category overlap $\kappa_{\mathrm{card}}$ and boundary noise $\kappa_{b}$ similar to the values in ref. 20. The encoding noise $\kappa_{i}$, the weight w of the categorical mismatch, and an additive motor noise $\kappa_{m}$ were adjusted so that the magnitude of the bias and SD matched the data in the baseline condition. We then fixed these common parameters and predicted behavior in the oriented surround condition (tilt illusion) based on the model, with the additional assumption that the surround orientation created an additional category boundary with relative sharp boundaries (high $\kappa_{\text{surr}}$) as the surround is always present. *SI Appendix*, Table S1 lists the values of all model parameters for simulating the tilt illusion.

### Voxel Encoding Model Based on Steerable Pyramid.

We estimated the changes in the measured encoding FI that arise only due to differences in stimulus configuration (i.e., nonoriented vs. oriented surround) in the absence of any potential change in neural response properties. We follow the approach of ref. 19 to create an image-computable model of voxel encoding (Fig. 7*A*). For a given stimulus image we use the steerable pyramid (33) to create filtered responses at different orientations (c) and spatial frequency (SF) bands (s). We used a complex pyramid and combined the real and imaginary parts to obtain single energy-like filter responses. This yielded multiple filtered images indexed by c and s: r(c,s). These images can be thought of as representing V1-like neuronal responses at every location of the visual space, each with a different orientation and spatial frequency selectivity.

To simulate voxel activity f(θ), we combined responses across orientation bands as $f_{s}\left(\theta \right)=\sum_{c}r\left(c,s\right)$. This produced a “retinotopic map” of voxel responses at different spatial scales. The encoding FI is defined as $J\left(\theta \right)=||df\left(\theta \right)/{d\theta ||}_{2}^{2}$, which is the FI assuming independent Gaussian response noise with unit variance for each voxel. In our case, a steerable pyramid with six orientation and six SF bands was constructed for each stimulus image using Pyrtools (79). We selected the SF band with the strongest orientation information based on the total FI (*SI Appendix*, Fig. S7), then applied the eccentricity-ROI analysis to compute FI at the center-surround boundary (*SI Appendix*, Fig. S8).

To compute the changes in FI, we first computed $J{\left(\theta \right)}_{\text{base}}$ using stimulus images with nonoriented surround. Note that $J{\left(\theta \right)}_{\text{base}}$ is nonzero, representing the vignetting effect reported by Roth et al. (19), and is also uniform, since any effect of stimulus configuration in the nonoriented condition is isotropic by construction. We then computed $J{\left(\theta \right)}_{\text{surr}}$ using stimuli with oriented surround. The change was calculated as $\Delta J\left(\theta \right)=J{\left(\theta \right)}_{\text{surr}}-J{\left(\theta \right)}_{\text{base}}$, and the results are shown in Fig. 7*B*. See *SI Appendix*, Figs. S7 and S8, and the associated text for a more extensive discussion on the issue of stimulus vignetting, including FI calculated separately at each spatial scale and eccentricity ROI.

## Supplementary Material

Appendix 01 (PDF)

## Data Availability

The fMRI and behavioral data have been deposited in Open Science Framework (https://osf.io/9uqbd/) (80). The software developed for data analysis is available through GitHub (https://github.com/lingqiz/orientation-encoding) (81). Previously published data were also used for this work (14).
