# Supplementary material for: The tilt illusion arises from an efficient reallocation of neural coding resources at the contextual boundary
Source: Proc Natl Acad Sci U S A. 2025 Apr 23;122(17):e2421565122. doi: 10.1073/pnas.2421565122 (PMC12054787; doi:10.1073/pnas.2421565122)
Supplement: Supplementary file 1 — Appendix 01 (PDF) [file pnas.2421565122.sapp.pdf]

Supporting information for "The tilt illusion arises from an efficient reallocation of neural coding resources at the contextual boundary"

Ling-Qi Zhang, Jiang Mao, Geoffrey K. Aguirre, and Alan A. Stocker

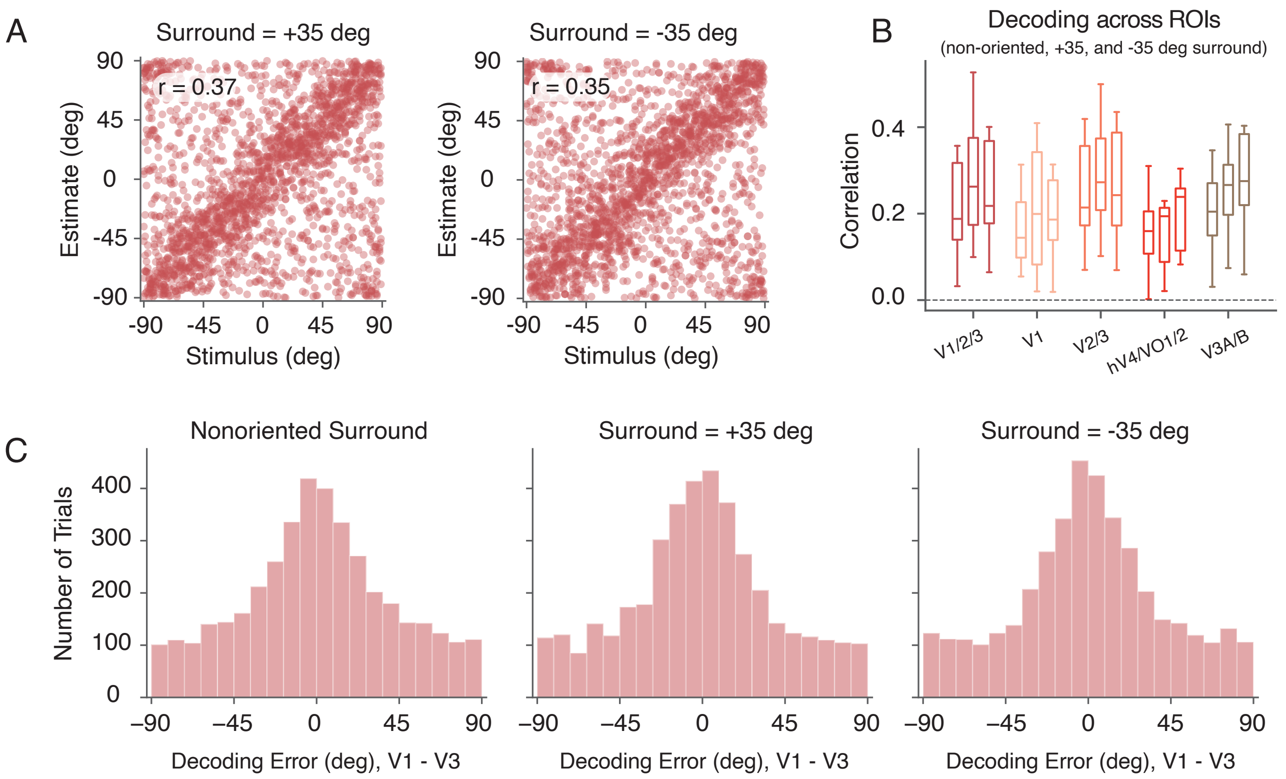

Supplementary Figure 1. Orientation decoding performance. **A)** Same as Fig. 2C, scatter plot of stimulus orientation (x-axis) versus decoded orientation (y-axis) from the early visual cortex (V1 to V3), and the two oriented surround ($\pm$ 35) conditions. **B)** Same as Fig. 2D, but with decoding correlation plotted separately for each of the three surround conditions within each ROI. Note the decoding performance r is quantified as the circular correlation between stimulus and the MLE estimates. **C)** Histogram of decoding errors from areas V1/2/3, for the combined subject across the three surround conditions.

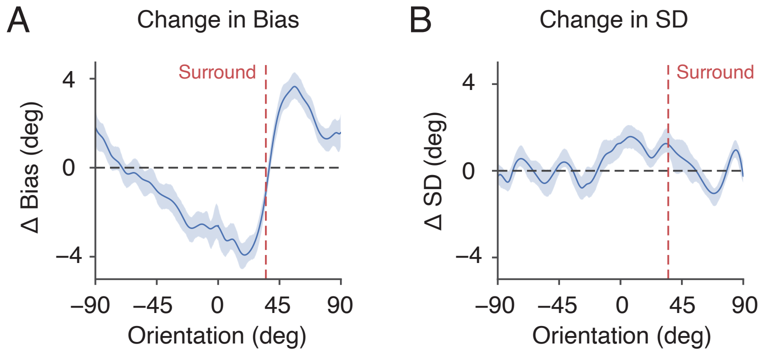

Supplementary Figure 2. Effect of surround modulation on orientation estimation. **A)** Difference in estimation bias between the nonoriented and the oriented surround condition. **B)** Difference in the standard deviation of the orientation estimates between the nonoriented and the oriented surround condition. Shaded areas indicate $\pm$SEM.

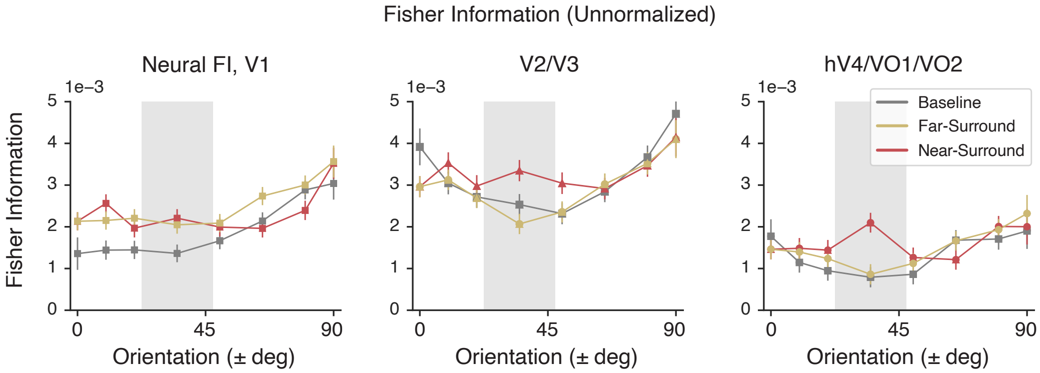

Supplementary Figure 3. Unnormalized Neural FI. Same data as in Fig. 4E, but plotting the unnormalized Fisher information instead. In terms of absolute effect size, V1 shows no significant effect (i.e., near- and far-surround conditions are similar). Between V2/V3 and hV4/VO1/2, the absolute effect size is comparable but slightly larger and more significant in hV4/VO1/2. However, because the FI in hV4/VO1/2 is lower in the nonoriented (baseline) condition, the relative effect size in these areas appears larger, with the surround effect doubling the amount of FI compared to the baseline for orientations close to the surround orientation.

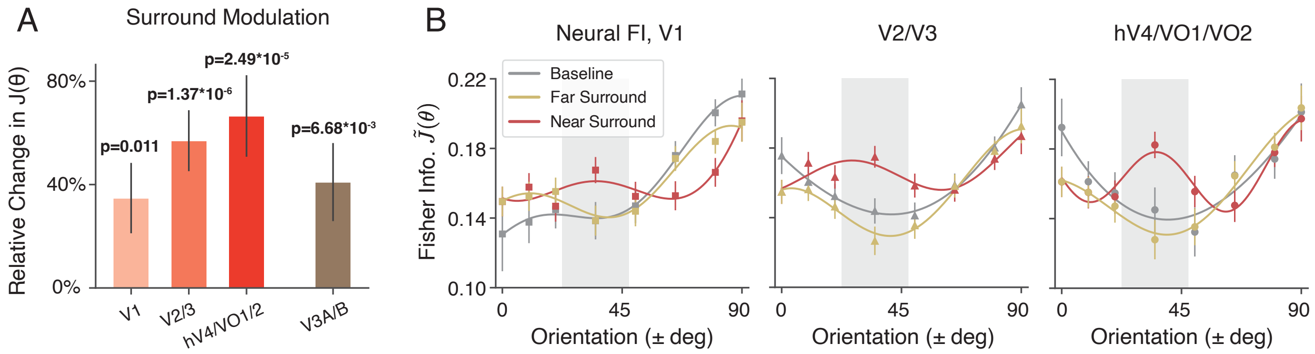

Supplementary Figure 4. Neural encoding across visual areas with expanded eccentricity ROI. We repeated the same analysis as in Figs. 4D, E but expanded the eccentricity selection to cover the entire stimulus (1 to 15 degrees). **A)** The relative change in neural FI with respect to the baseline near the surround orientation across different visual cortex ROIs. **B)** Comparison of neural FI along the visual ventral stream, between near-surround side and far-surround side orientations in the oriented surround condition, and the baseline condition. Error bars indicate $\pm$SEM.

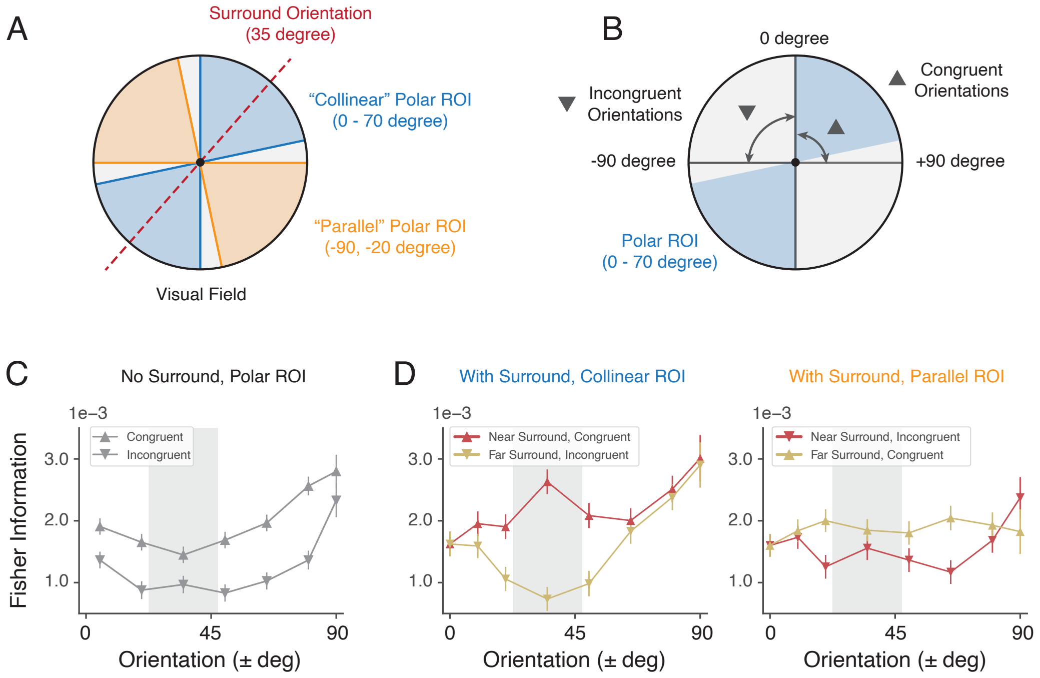

Supplementary Figure 5. Neural FI for different voxel polar angle ROIs. In Fig. 5, we examined the effect of surround modulation for voxels at different visual eccentricities. Here, we conduct a similar analysis but based on the polar angle assignment of the voxels. **A)** Two separate polar angle ROIs for the V2/V3 area were constructed based on the voxel alignment with the surround orientation. The "collinear" ROI is defined by selecting voxels within the polar angle range that align with the surround orientation; The "parallel" ROI is defined by voxels in the range orthogonal to the surround orientation. Note that a symmetrical construction was applied for the -35 degree surround orientation. **B)** For any polar ROI, center orientations aligned with the voxel ROI's polar angle range are considered congruent, while center orientations outside the voxel ROI's polar angle range are considered incongruent. For the baseline condition (nonoriented surround), only the congruent and incongruent categories are applicable. For the oriented surround conditions, voxels can be congruent and collinear, parallel and incongruent, and vice-versa. **C)** Neural FI in the baseline condition, plotted separately for the orientation range that is congruent with the polar angle ROI range, and the orientation range that is incongruent. We see the same general pattern of a relative increase in FI for the cardinal orientations (as seen in Fig. 3D). However, we also find an overall increase in FI for the congruent orientation range and a corresponding decrease for the incongruent orientations. This pattern is reminiscent of the radial bias previously observed in voxel orientation tuning measurements (80). **D)** Neural FI in the oriented surround condition for the collinear ROI (left) and the parallel ROI (right). To properly interpret the FI curves in the oriented surround conditions, however, we need to compare FI to its corresponding congruent or incongruent baseline. In the collinear ROI, we observe that the presence of the surround increases encoding FI near the surround orientation (relative to the congruent baseline). In the parallel ROI, the surround effect is significant but less pronounced (compared to the incongruent baseline). In either case, we do not see a strong effect for far-surround orientations.

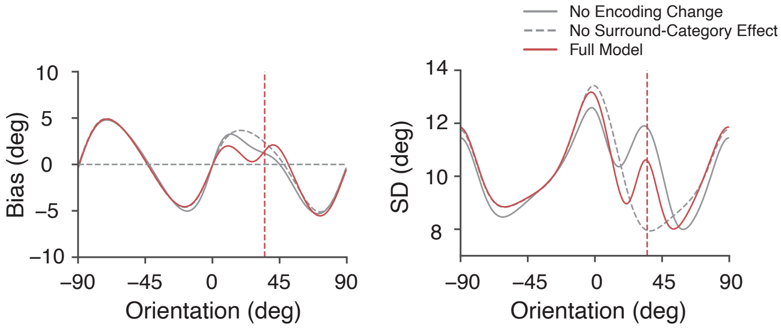

Supplementary Figure 6. Both the surround-induced change in sensory encoding and the categorical boundary at the surround orientation are necessary for a correct Bayesian observer model prediction of the tilt illusion. Panels show the predicted estimation bias and standard deviation of the model in the surround condition: Solid gray lines represent the model prediction without assuming a change in sensory encoding (i.e., using the encoding pattern from the baseline condition), while the dashed gray lines represent the model prediction without assuming the categorical boundary at the surround orientation. The solid red lines represent the prediction based on the full model (same as in Fig. 6). Both mechanisms are required to correctly predict the characteristic repulsive bias in the tilt illusion.

Parameter	Value
κ_i: sensory noise	10.5
κ_b: boundary noise	60
κ_{card}: cardinal category overlap	2
κ_{surr}: surround category overlap	24
w: categorical weight	0.72
κ_m: motor noise	48

Supplementary Table 1. Parameters used for the simulations of the Bayesian observer model shown in Fig. 6 (See *Methods* for a detailed description of the model).

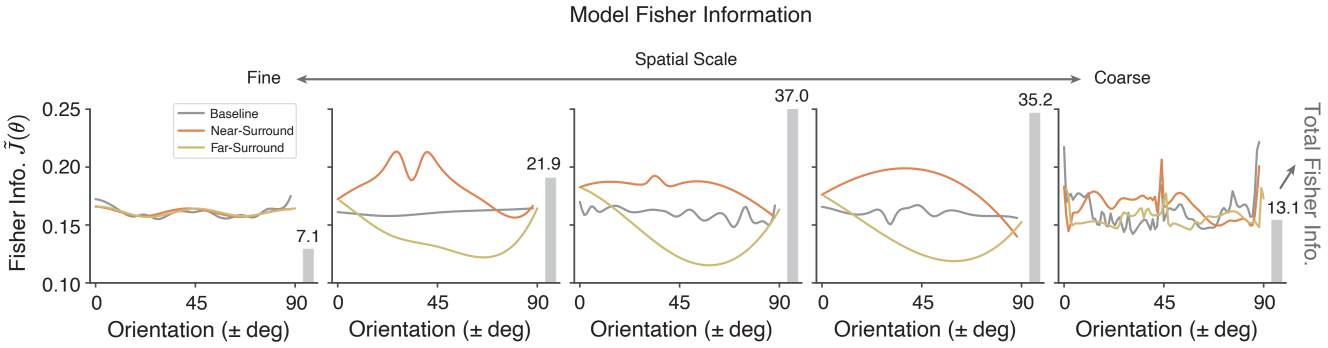

Supplementary Figure 7. The neural basis of orientation decoding using functional imaging has been the subject of ongoing debate (18, 19, 81), with recent findings (known as stimulus vignetting) challenging the notion that decoding is based on sensitivity to columnar-level neural tuning. In one sense, our results are independent of the outcome of this debate: Our neural measures of orientation encoding show strong consistency with behavioral data, indicating that regardless of the precise source of the orientation signal, it is indeed utilized by downstream processes and reflected in behavior. Regardless, following the approach of (19), we calculated the neural FI of the voxel encoding model using a steerable pyramid decomposition across different spatial scales. At each spatial scale, a single response map was obtained by averaging across orientation channels. The gray bar in each panel represents the total FI, which quantifies the strength of orientation information due to vignetting. We found that the middle spatial scale exhibited the strongest effect in our model. However, all three spatial scales displayed qualitatively similar patterns. The lowest and highest spatial scales showed no effect of surround modulation.

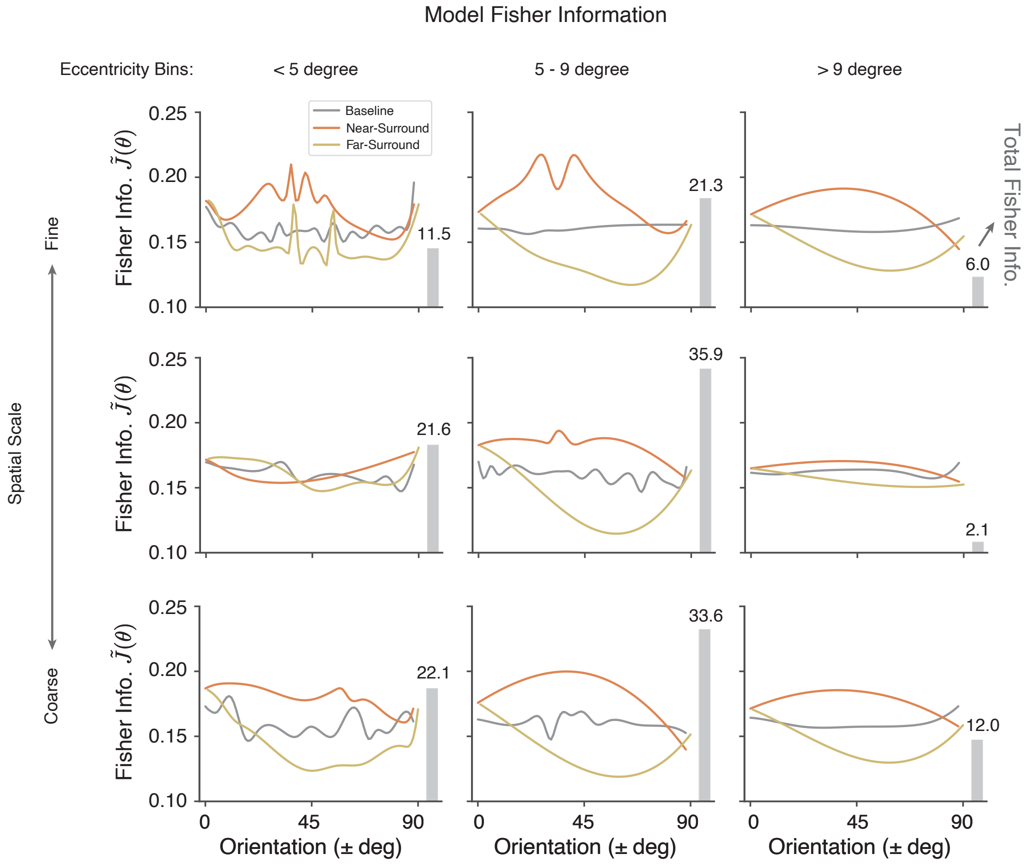

Supplementary Figure 8. We further analyzed the steerable pyramid model by using the eccentricity-ROI approach we used for our data. The encoding FIs in all three spatial scales are qualitatively similar at the stimulus boundary (5 - 9 degree eccentricity): flat for the baseline, with a broad increase for near-surround orientations, and a broad decrease for far-surround orientations. We think there are four aspects of our data that are inconsistent with this "vignetting only" model. First, we observed an anisotropy in orientation encoding under the non-oriented surround condition. Given that the stimuli were designed to be isotropic (gray line), this effect must arise from anisotropies inherent in the neural representation of orientation. Second, our eccentricity ROI analysis showed comparable decoding performance from voxels away from the stimulus boundary (in the center region) and those at the stimulus boundary. Third, we found that the effects of stimulus configuration in the oriented surround condition are broad and symmetrical at the surround orientation and orientations orthogonal to it (orange and yellow line), inconsistent with the local changes we observed in our data. Finally, the model fails to replicate the increased effects of surround modulation across the visual hierarchy.

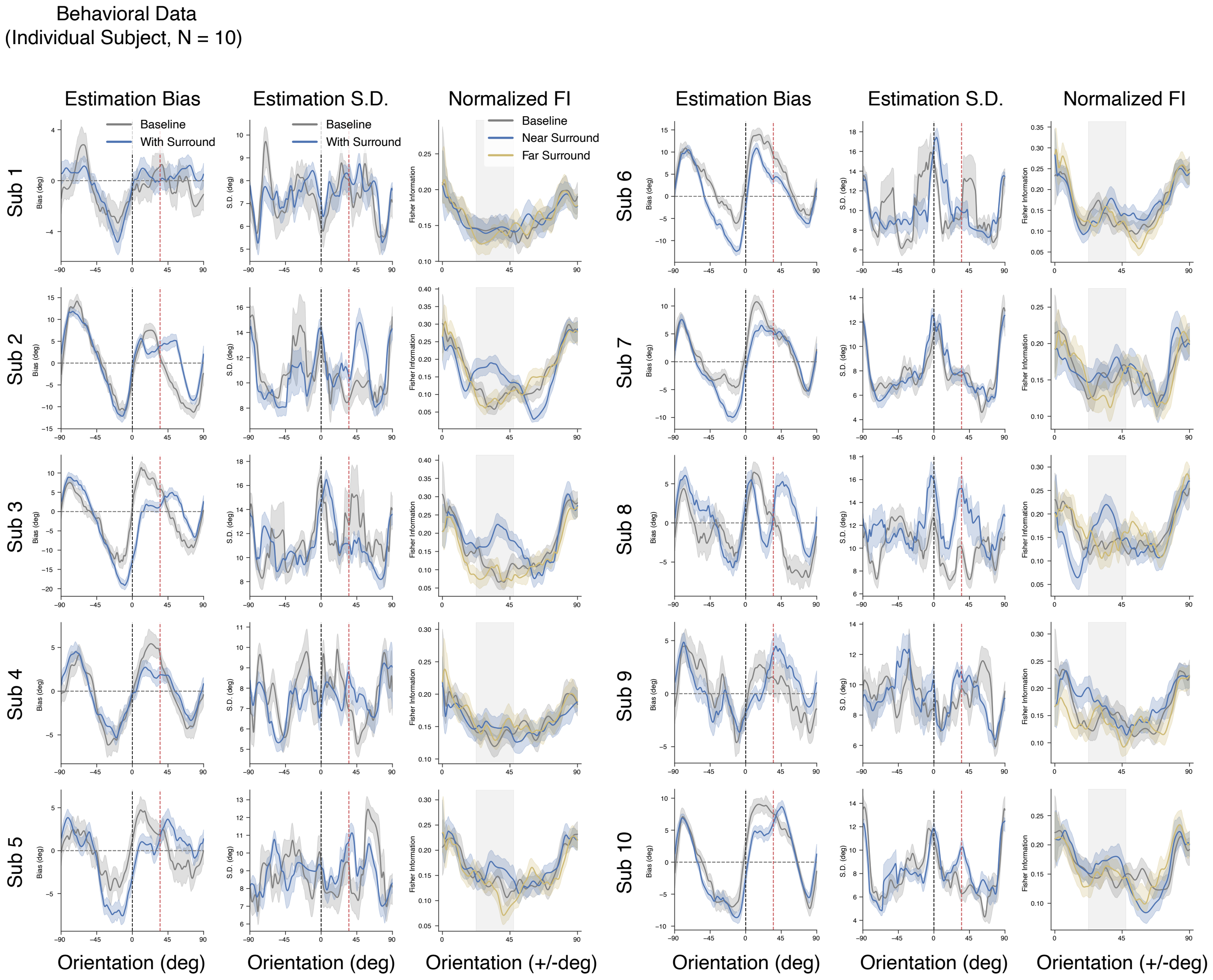

Supplementary Figure 9. The bias and standard deviation of the orientation estimates, and the normalized behavioral FI for individual subjects (N=10).

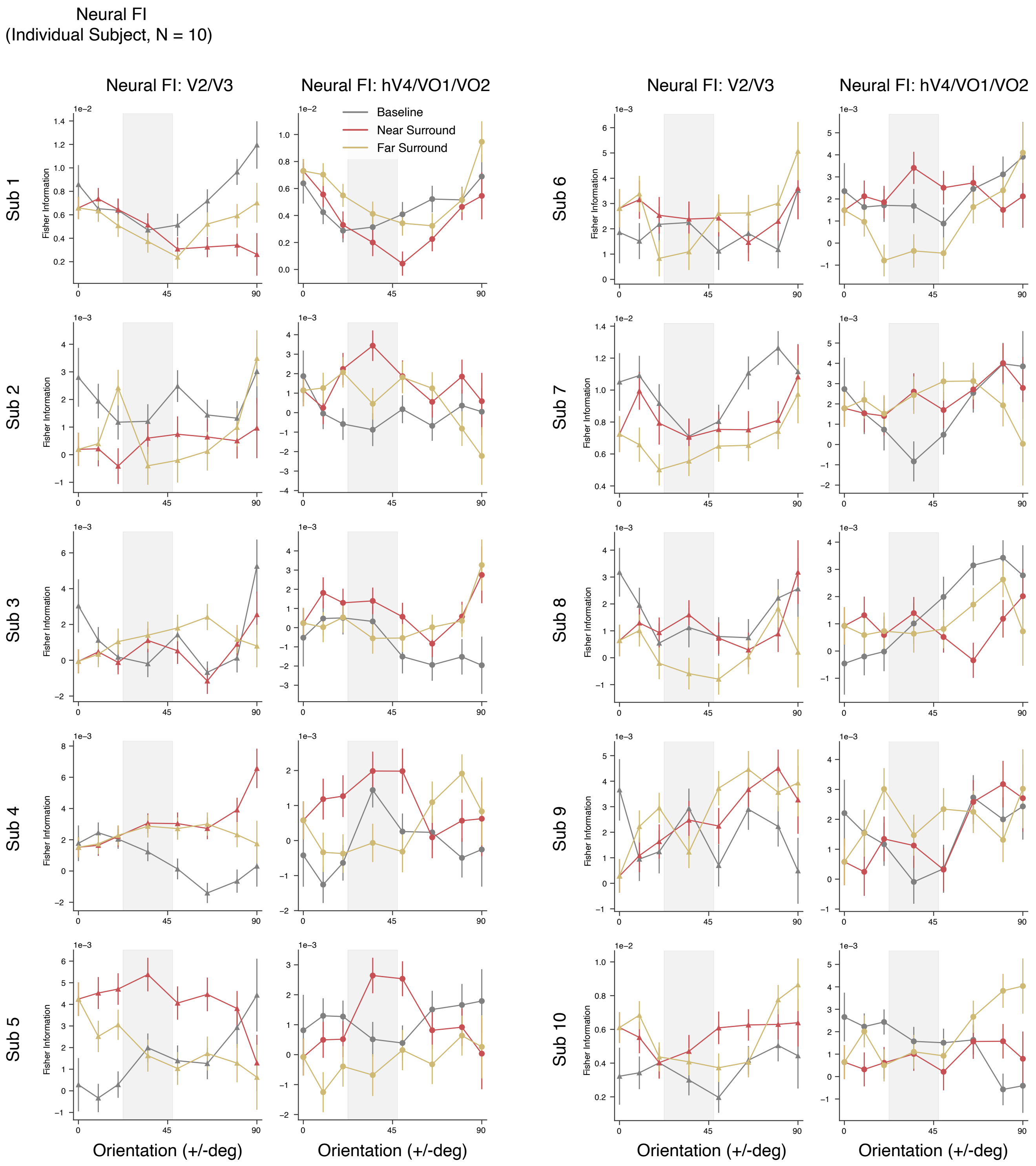

Supplementary Figure 10. The unnormalized neural FI from two visual area ROIs (between 1 - 7 degrees for V2/V3 and hV4/VO1/2) for individual subjects (N=10).
